# Renal Tubule-Specific Deletion of Nephrocystin 3 *(Nphp3)* Causes Infantile Nephronophthisis-like Phenotypes in Mice

**DOI:** 10.3390/ijms27062687

**Published:** 2026-03-15

**Authors:** Xuanjin Du, Chunyan Wang, Ye Fang, Gangqi Wang, Yihui Zhai, Qian Shen, Xiaoshan Tang, Hong Xu

**Affiliations:** 1 Department of Nephrology, Children’s Hospital of Fudan University, National Children’s Medical Center, Shanghai 201102, China; 2 Shanghai Kidney Development and Pediatric Kidney Disease Research Center, Shanghai 201102, China; 3 Children’s Hospital of Fudan University, Institutes of Biomedical Sciences, Fudan University, Shanghai 200032, China; 4 National Key Laboratory of Kidney Diseases, Beijing 100853, China

**Keywords:** nephrocystin 3 *(Nphp3)*, mouse model, renal cysts, renal fibrosis

## Abstract

Patients with nephronophthisis caused by nephrocystin 3 *(NPHP3)* variants rapidly progress to end-stage kidney disease. However, existing *Nphp3* mouse models fail to fully recapitulate the characteristics of this disease. We generated a renal tubule-specific *Nphp3* knockout mouse model that more accurately mirrors the human disease course. The mouse model was first validated by confirming the loss of Nphp3 protein expression in renal tubules. Comprehensive phenotypic analyses were then performed to assess both renal and extrarenal manifestations. The origin of renal cysts was investigated, and the underlying mechanisms were further validated. We successfully generated a renal tubule-specific *Nphp3* knockout mouse model (*Cdh16-Cre*; *Nphp3^flox/flox^*). These mice exhibited a markedly shortened lifespan (5–8 weeks) and developed key features of infantile nephronophthisis, including early-onset renal cysts originating from distal tubules and collecting ducts, progressive interstitial fibrosis that was evident by postnatal week 2, a rapid decline in kidney function, and increased urinary protein levels. Importantly, treatment with the vasopressin V2 receptor antagonist tolvaptan or the mitogen-activated extracellular signal-regulated kinase (MEK) inhibitor 2-(2-chloro-4-iodoanilino)-N-(cyclopropylmethoxy)-3,4-difluorobenzamide (CI-1040) significantly attenuated cyst growth and improved kidney morphology, confirming shared pathogenic pathways with other *Nphp3* models. We established a renal tubule-specific *Nphp3* knockout mouse model that accurately recapitulates the aggressive infantile form of nephronophthisis characterized by early cystogenesis, progressive fibrosis, and a shortened lifespan, and is ideal for evaluating novel interventions against this currently untreatable ciliopathy.

## 1. Introduction

Nephronophthisis-related ciliopathies (NPHP-RCs) are characterized by progressive cystic kidney disease and constitute a group of autosomal recessive cystic kidney disorders [1]. NPHP-RC is the major cause of end-stage kidney disease (ESKD) in childhood or adolescence [2]. To date, more than 90 genes have been shown to cause renal ciliopathies [3]. Among them, nephrocystin 1 *(NPHP1)* and nephrocystin 3 *(NPHP3)* are the most common pathogenic genes and are carried by 58.3% of Chinese children with NPHP-RC. *NPHP3*-related nephronophthisis represents one of the more aggressive forms and is often associated with an earlier onset and rapid progression to ESKD and liver function abnormalities [4]. Simultaneously, *NPHP1* (20–25%) [5] and *NPHP3* (12–16%) [6] are recognized as the primary causative genes of NPHP-RC on a global scale [7].

*NPHP3* encodes a 1330-amino acid protein that is widely expressed in kidney tubules, retina, respiratory epithelium, liver, biliary tract and neural tissues [7]. Systemic knockout of *Nphp3* leads to embryonic lethality [8]. Mutations in *NPHP3* are the cause of nephronophthisis in humans and polycystic kidney disease in mice with homozygous mutations (pcy) [9]. Recent studies suggest that NPHP3 deficiency disrupts ciliary stability and planar cell polarity in zebrafish embryos and *Xenopus laevis* [10]. Functionally, NPHP3 interacts with inversin (INVS) and suppresses INVS-mediated canonical wingless/integrated (Wnt) signaling [8]. However, the precise molecular cascade triggered by tubular epithelial-specific *NPHP3* inactivation, particularly in postnatal kidney homeostasis, remains incompletely defined.

Cyst formation in individuals with *NPHPs* is increasingly recognized as a consequence of disrupted tubule ciliary signaling, aberrant epithelial proliferation, and defective apoptotic regulation [3]. Studies have revealed that several pathways, including the Wnt, mechanistic target of rapamycin (mTOR), and cyclic adenosine monophosphate (cAMP) pathways, are involved in cystogenesis, suggesting potential intervention targets [11,12]. Several therapeutic interventions show promise, including the use of vasopressin receptor 2 antagonists (e.g., tolvaptan), cyclin-dependent kinase inhibitors (e.g., roscovitine), Hedgehog agonists (e.g., purmorphamine), and mTOR inhibitors (e.g., rapamycin) [3].

In our study, we constructed a mouse model in which *Cdh16-Cre*; *Nphp3^flox/flox^*-targeted deletion of Nphp3 in renal tubular epithelial cells resulted in the development of renal characteristics of infantile nephronophthisis observed in humans. In addition, we evaluated the therapeutic potential of tolvaptan and 2-(2-chloro-4-iodoanilino)-N-(cyclopropylmethoxy)-3,4-difluorobenzamide (CI-1040) in slowing cyst progression in this mouse model, further confirming the consistency of its pathogenic mechanisms with those of *Nphp3^pcy/pcy^* mice. We generated a novel mouse model with renal tubular epithelial-specific knockout of *Nphp3* that represents a new biological tool for investigating this rapidly progressive form of nephronophthisis.

## 2. Results

### 2.1. Mouse Model Validation and Phenotyping

#### 2.1.1. Generation and Validation of Renal Tubule-Specific *Nphp3* Knockout Mouse Model

**Figure 1:**
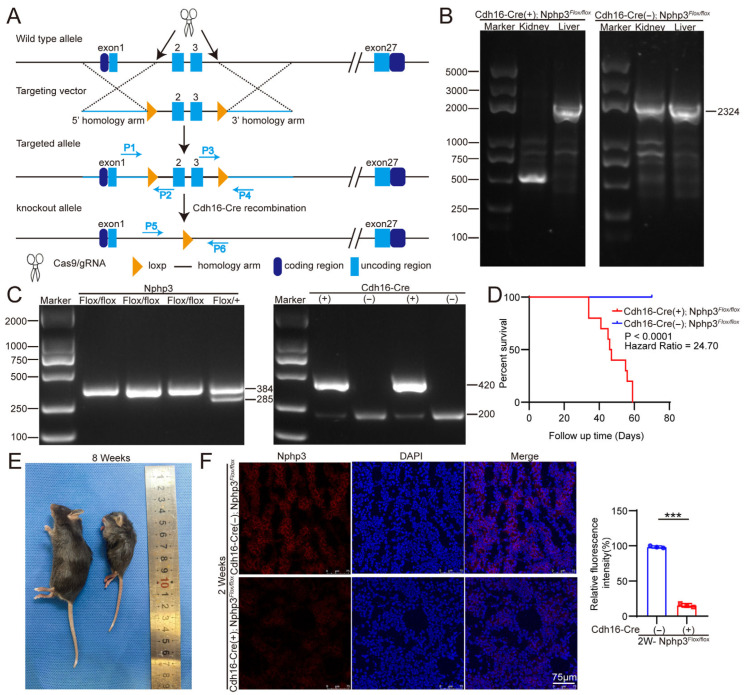
Construction of a renal tubule-specific nephrocystin 3 *(Nphp3)* gene knockout mouse model. (**A**) Schematic diagram illustrating the strategy used to generate renal tubule-specific knockout mice with a deletion of exons 2–3 of the *Nphp3* gene. Genotyping was performed using primers P1–P6. Blue squares represent exons, and arrows indicate the sites of action. (**B**) Results of the Cre recombinase activity assay. Cre activity was detected specifically in the kidneys of *Cdh16-Cre*; *Nphp3^flox/flox^* mice. (**C**) Representative genotyping results. The left panel shows the genotyping results for the floxed allele, and the right panel shows the results for the Cre transgene. (**D**) Kaplan—Meier survival curves of the mice. *Cdh16-Cre*; *Nphp3^flox/flox^* mice exhibited a median survival time of 5–8 weeks (*Cdh16-Cre*; *Nphp3^flox/flox^* mice, *n* = 10; *Nphp3^flox/flox^* mice, *n* = 11). (**E**) Image displaying the gross morphology of 8-week-old mice. The left panel shows an *Nphp3^flox/flox^* mouse, while the right panel shows a *Cdh16-Cre*; *Nphp3^flox/flox^* mouse. The mouse on the right exhibits a significantly emaciated physique, whereas the control mouse on the left appears normal. (**F**) Immunofluorescence staining for Nphp3 in mouse kidney sections. Blue fluorescence indicates nuclei stained with 4′,6-diamidino-2-phenylindole (DAPI), while red fluorescence shows Nphp3 protein. A significant decrease in Nphp3 expression was observed in the renal tubules of *Cdh16-Cre*; *Nphp3^flox/flox^* mice (*n* = 3 mice/group). The log-rank test was used to analyze the survival outcomes. Statistical significance was defined as *** *p* < 0.001 using an unpaired two-tailed Student’s *t*-test for two-group comparisons.

The strategy used to generate the mouse model in this study is illustrated in Figure 1A. Exon 1 was excluded because of gene overlap, and exons 2–3 were deleted to silence the expression of the *Nphp3* gene. Model mice were identified through genotyping. Genotyping confirmed that the *Cdh16-Cre*; *Nphp3^flox/flox^* mice were homozygous for the floxed allele and positive for Cre (Figure 1C). Furthermore, the results of the Cre activity assays verified that Cre recombinase activity was exclusively detected in the kidneys of *Cdh16-Cre*; *Nphp3^flox/flox^* mice (Figure 1B). Immunofluorescence staining subsequently showed the absence of NPHP3 protein expression in the renal tubules of *Cdh16-Cre*; *Nphp3^flox/flox^* mice (Figure 1F). The Western blot results are not presented herein due to the poor specificity of the antibody. Collectively, these results indicate the successful generation of a renal tubule-specific *Nphp3* knockout mouse model.

Compared with wild-type mice, *Cdh16-Cre*; *Nphp3^flox/flox^* mice exhibited a significantly shortened lifespan, surviving only 5 to 8 weeks, whereas no such difference was observed in control mice (*Nphp3^flox/flox^*) (Figure 1D). The sex distribution of *Cdh16-Cre*; *Nphp3^flox/flox^* mice was not significantly different (28 females: 30 males). The gross morphology of 8-week-old mice is shown in Figure 1E. The left panel shows an *Nphp3^flox/flox^* mouse, while the right panel shows a *Cdh16-Cre*; *Nphp3^flox/flox^* mouse. The mouse on the right exhibits a significantly emaciated physique, whereas the control mouse on the left appears normal.

#### 2.1.2. *Cdh16-Cre*; *Nphp3^flox/flox^* Mice Exhibited Renal Cyst Formation, Tubulointerstitial Fibrosis, and Reduced Renal Function

**Figure 2:**
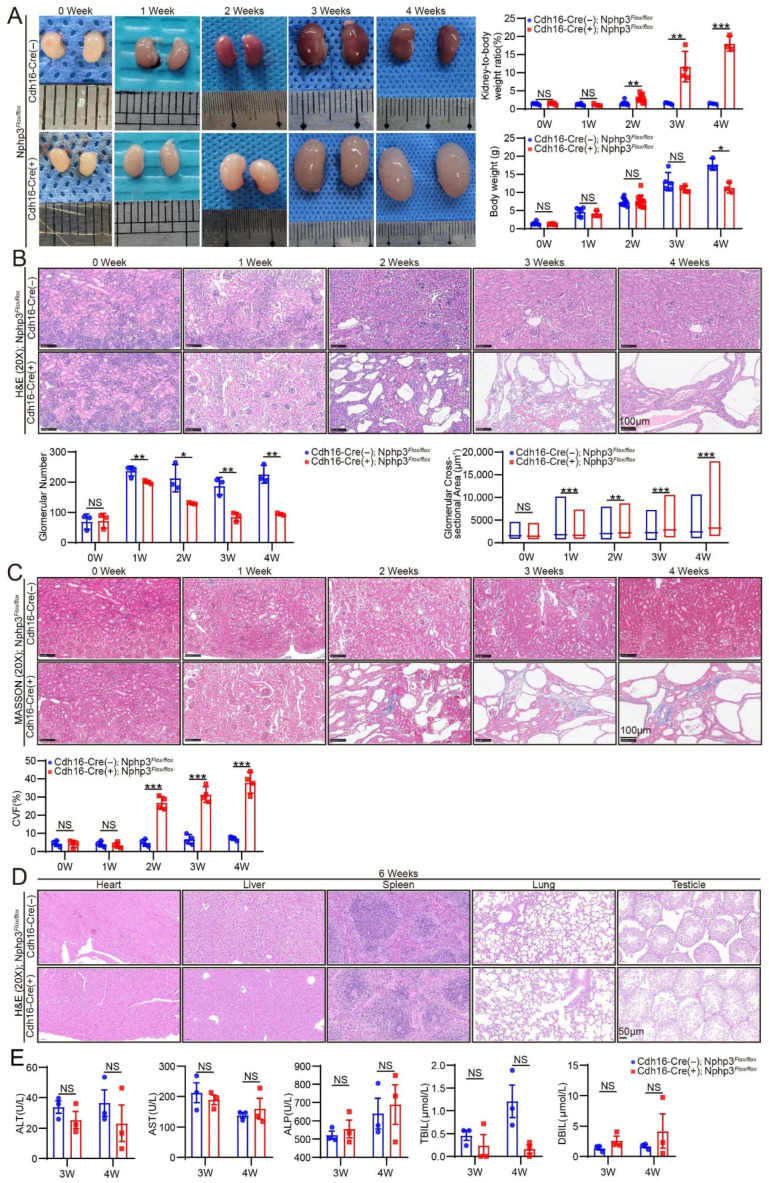
Renal cystic and fibrotic phenotypes in *Cdh16-Cre*; *Nphp3^flox/flox^* mice with no significant abnormalities in other major organs. (**A**) Gross images of kidneys from mice aged 0 to 4 weeks (0–4 W), along with body weight and kidney weight-to-body weight ratio (KW/BW) for mice aged 0–4 W (*n* ≥ 3 mice/group). (**B**) Representative hematoxylin and eosin (H&E)-stained kidney sections (20×) from 0–4-week-old mice. The quantitative analysis shows the glomerular count and glomerular cross-sectional area of the largest kidney section (*n* = 3 mice/group). (**C**) Masson’s trichrome-stained kidney sections (20×) and corresponding quantitative analysis of the collagen volume fraction (CVF) in mice aged 0–4 W (*n* ≥ 3 mice/group). (**D**) H&E-stained sections (20×) of major organs (heart, liver, spleen, lung, and testis) from 6-week-old mice. No significant pathological abnormalities were observed in these organs of *Cdh16-Cre*; *Nphp3^flox/flox^* mice. (**E**) Serum levels of markers of liver function, including alanine aminotransferase (ALT), aspartate aminotransferase (AST), alkaline phosphatase (ALP), total bilirubin (TBIL), and direct bilirubin (DBIL) levels (*n* = 3 mice/group). Statistical significance was defined as * *p* < 0.05; ** *p* < 0.01; *** *p* < 0.001; and *p* > 0.05 (NS [not significant]) using an unpaired two-tailed Student’s *t*-test for two-group comparisons.

*Cdh16-Cre*; *Nphp3^flox/flox^* mice exhibit a renal phenotype that closely recapitulates that of humans with *NPHP3*-associated nephronophthisis, and is characterized by early-onset cystogenesis, progressive tubulointerstitial fibrosis, and a consequent decline in kidney function. Images of the gross kidney morphology and stained kidney sections from mice at 0 weeks to 4 weeks (W) are shown in Figure 2. Renal cysts in *Cdh16-Cre*; *Nphp3^flox/flox^* mice began to form at 1 week, after which they progressively enlarged and compressed the surrounding renal parenchyma. An examination of the gross morphology revealed that the kidneys of *Cdh16-Cre*; *Nphp3^flox/flox^* mice were enlarged and paler than those of control mice (*Nphp3^flox/flox^*). The kidney weight-to-body weight ratio (K/W) of *Cdh16-Cre*; *Nphp3^flox/flox^* mice was also significantly higher than that of control mice starting at 2 W. While no significant difference in body weight was observed between the groups from 0 to 3 weeks, the body weight of *Cdh16-Cre*; *Nphp3^flox/flox^* mice failed to increase significantly from 3 to 4 weeks. Consequently, the body weight of *Cdh16-Cre*; *Nphp3^flox/flox^* mice was significantly lower than that of control mice at 4 weeks (Figure 2A,B).

**Figure 3:**
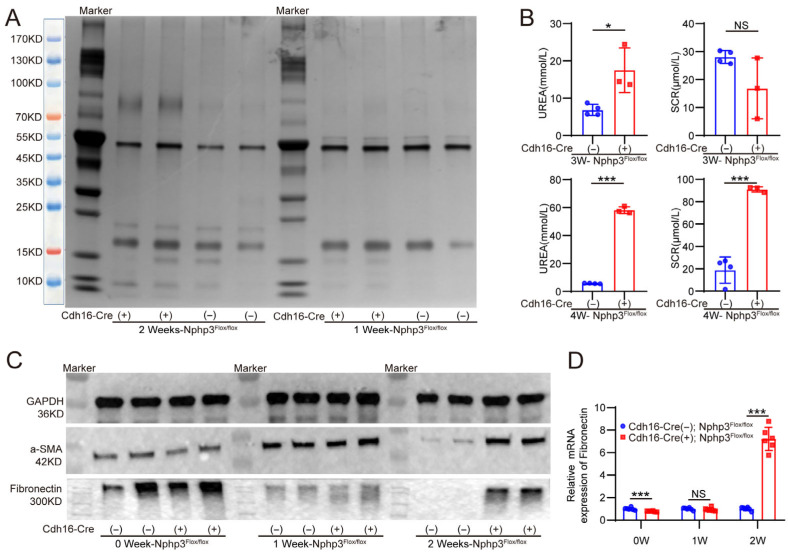
Impaired renal function and upregulation of fibrosis markers in 2-week-old *Cdh16-Cre*; *Nphp3^flox/flox^* mice. (**A**) Silver staining of urinary proteins. The total urinary protein content was significantly increased in *Cdh16-Cre*; *Nphp3^flox/flox^* mice compared with *Nphp3^flox/flox^* mice at 2 weeks of age. Urinary protein molecular weight reference: use the left-side marker as a size reference. (**B**) Serum levels of markers of renal function, including serum creatinine (SCR) and serum urea (UREA) levels (*n* ≥ 3 mice/group). (**C**,**D**) Expression levels of renal fibrosis markers. Protein and messenger RNA (mRNA) expression of key fibrotic indicators in the kidneys of *Cdh16-Cre*; *Nphp3^flox/flox^* mice (*n* ≥ 3 mice/group). Statistical significance was defined as * *p* < 0.05; *** *p* < 0.001; and *p* > 0.05 (NS [not significant]) using an unpaired two-tailed Student’s *t*-test for two-group comparisons.

**Figure 4:**
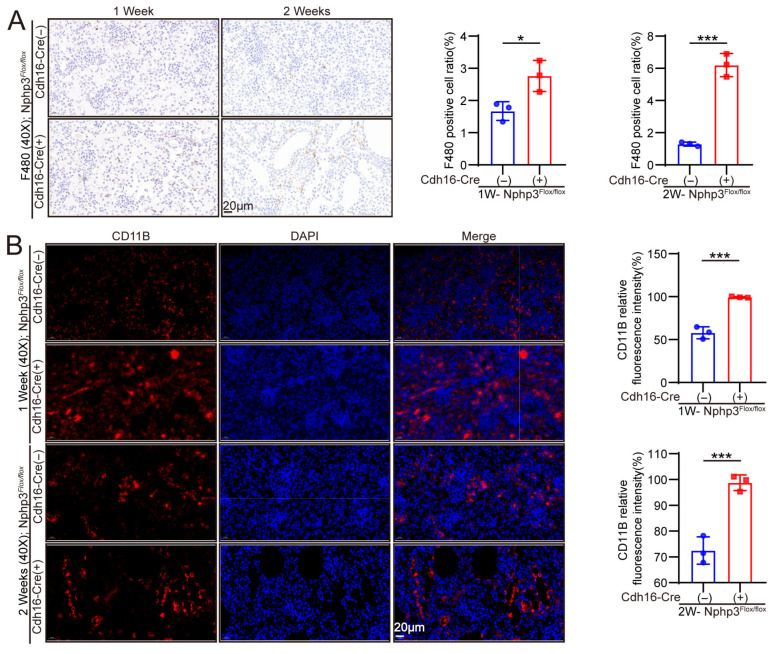
Inflammatory cell aggregates were already present in the kidneys of 1-week-old *Cdh16-Cre*; *Nphp3^flox/flox^* mice. (**A**) F4/80 immunohistochemistry staining reflected the state of macrophage infiltration. The image showed significant macrophage infiltration in the kidneys of 1-week-old *Cdh16-Cre*; *Nphp3^flox/flox^* mice compared to control mice (*n* = 3 mice/group). (**B**) Integrin alpha-M (CD11B) immunofluorescence staining reflected the state of neutrophil infiltration. The image showed significant neutrophil infiltration in the kidneys of 1-week-old *Cdh16-Cre*; *Nphp3^flox/flox^* mice compared to control mice (*n* = 3 mice/group). Blue fluorescence represents DAPI-stained nuclei, and red indicates CD11B protein. Statistical significance was defined as * *p* < 0.05 and *** *p* < 0.001 using an unpaired two-tailed Student’s *t*-test for two-group comparisons.

Quantification of the number of glomeruli and the cross-sectional area of the largest kidney section revealed no significant differences between *Cdh16-Cre*; *Nphp3^flox/flox^* and control mice at 0 W. However, from 1 week to 4 weeks, *Cdh16-Cre*; *Nphp3^flox/flox^* mice exhibited a significant decrease in the glomerular number. With respect to the glomerular area, a relative decrease was observed at 1 W, followed by a relative increase from 2 W to 4 W (Figure 2B). Masson’s trichrome staining of kidney sections and the subsequent quantification of the collagen volume fraction (CVF) revealed evident fibrosis in *Cdh16-Cre*; *Nphp3^flox/flox^* mice as early as 2 W, with the severity of renal fibrosis progressively increasing from 2 W to 4 W (Figure 2C). Consistent with these findings, Western blot and quantitative real-time reverse transcription polymerase chain reaction (qRT-PCR) analyses revealed the significant upregulation of both the protein and mRNA levels of fibrosis markers (fibronectin and alpha-smooth muscle actin[α-SMA]) in the model mice starting at 2 W (Figure 3C,D). In addition, immunostaining was performed on kidney tissues from 1 and 2-week-old mice to assess immune cell infiltration. These results revealed that inflammatory cell infiltration was already present in the kidneys of *Cdh16-Cre*; *Nphp3^flox/flox^* mice as early as 1 week of age (Figure 4A,B).

Furthermore, significantly elevated serum creatinine and urea levels were observed in *Cdh16-Cre*; *Nphp3^flox/flox^* mice compared with those in control mice at 3 W and 4 W (Figure 3B). Silver staining of randomly collected urine samples revealed a significant increase in total urinary protein levels in *Cdh16-Cre*; *Nphp3^flox/flox^* mice compared with that in *Nphp3^flox/flox^* mice at 2 W (Figure 3A). These results indicate the onset of impaired renal function in *Cdh16-Cre*; *Nphp3^flox/flox^* mice by 2 weeks of age.

#### 2.1.3. *Cdh16-Cre*; *Nphp3^flox/flox^* Mice Exhibited No Extrarenal Abnormalities

We performed hematoxylin and eosin (H&E) staining of tissue sections from the heart, liver, spleen, lung, and reproductive system of 6-week-old *Cdh16-Cre*; *Nphp3^flox/flox^* mice to assess the phenotypes of other major organs. At this end-stage survival time point, no abnormalities were observed in these organs compared with those in *Nphp3^flox/flox^* control mice (Figure 2D). These findings were corroborated by serum biochemical analyses, which showed no significant differences in liver function parameters (alanine aminotransferase [ALT], aspartate aminotransferase [AST], alkaline phosphatase [ALP], total bilirubin [TBIL], or direct bilirubin [DBIL]) between *Cdh16-Cre*; *Nphp3^flox/flox^* and control mice at 3–4 weeks of age, indicating normal liver function in the mutant mice.

### 2.2. Exploration of the Origin and Mechanisms Behind Cyst Formation

#### 2.2.1. Renal Cysts Originate from the Distal Tubules and Collecting Ducts

**Figure 5:**
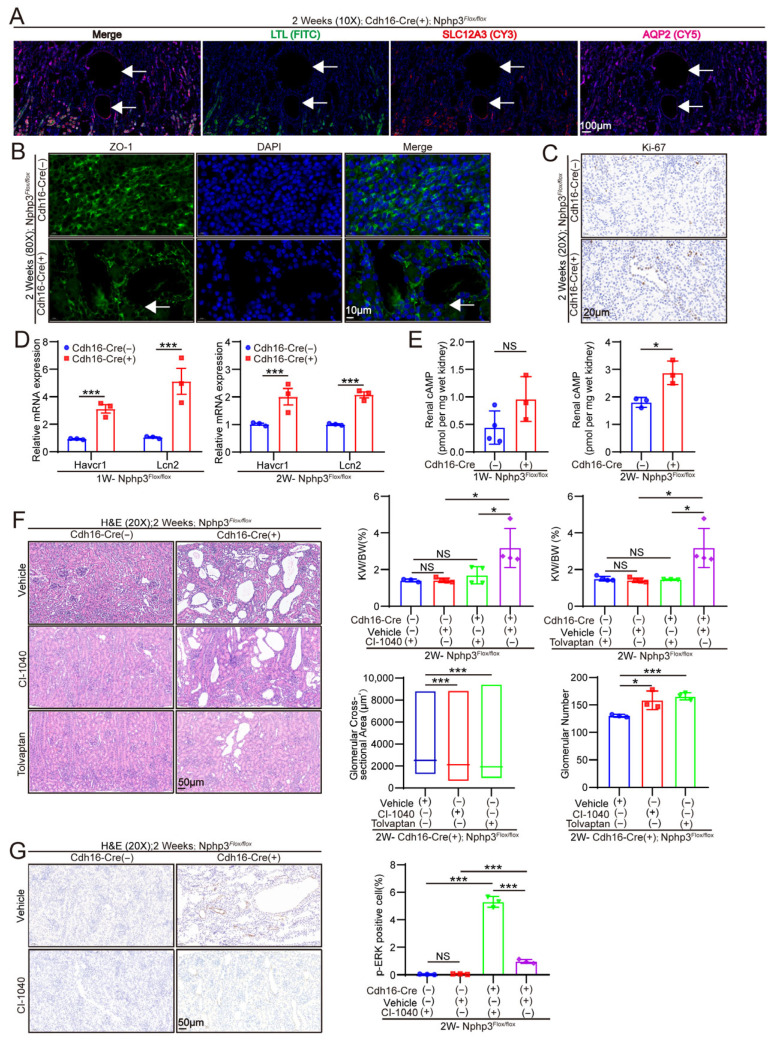
Renal cysts in *Cdh16-Cre*; *Nphp3^flox/flox^* mice originate from the distal tubules and collecting ducts and can be rescued by tolvaptan and 2-(2-chloro-4-iodoanilino)-N-(cyclopropylmethoxy)-3,4-difluorobenzamide (CI-1040) treatment. (**A**) Immunofluorescence staining of kidneys from *Cdh16-Cre*; *Nphp3^flox/flox^* mice showing the staining of proximal tubules (LTL-FITC), distal convoluted tubules (SLC12A3-CY3), and collecting ducts (AQP2-CY5). These results indicate that renal cysts in these mice originate from the distal tubules and collecting ducts. White arrows indicate renal cysts. (**B**) Zonula occludens-1(ZO-1) immunofluorescence staining of the kidneys of 2-week-old mice, showing changes in epithelial cell polarity and disruption of epithelial integrity in the tubular walls, particularly the cyst walls, of *Cdh16-Cre*; *Nphp3^flox/flox^* mice, as indicated by the white arrows (*n* = 3 mice/group). The blue fluorescence indicates cell nuclei stained with DAPI, while the green fluorescence shows the ZO-1 protein. (**C**) In 2-week-old *Cdh16-Cre*; *Nphp3^flox/flo^^x^* mice, renal tubular epithelial cells (particularly cyst-lining cells) exhibited a significant increase in Ki-67 positivity compared to control mice. (**D**) The gene expression levels of renal tubular injury markers (kidney injury molecule-1[KIM-1] and neutrophil gelatinase-associated lipocalin [NGAL]) were examined in 1 and 2-week-old mice. The results show that the expression of these tubular injury markers was significantly upregulated in *Cre*; *Nphp3^flox/flo^^x^* mice (*n* = 3 mice/group). (**E**) The cyclic adenosine monophosphate (cAMP) content per unit weight of kidney tissue was measured in 1 and 2-week-old mice. The results show a certain degree of increase in cAMP levels in the kidneys of *Cdh16-Cre*; *Nphp3^flox/flo^^x^* mice at 1 week of age, and this increase became more pronounced and statistically significant at 2 weeks (*n* ≥ 3 mice/group). (**F**) H&E-stained kidney sections (20×) from *Cdh16-Cre*; *Nphp3^flox/flox^* mice treated with tolvaptan or CI-1040. Compared with the vehicle control treatment, the administration of tolvaptan (5 mg/kg) or CI-1040 (10 mg/kg) significantly reduced renal cyst formation. In *Cdh16-Cre*; *Nphp3^flox/flox^* mice, treatment with either tolvaptan or CI-1040 significantly decreased the kidney KW/BW ratio, increased the number of glomeruli per maximal renal cross-section, and reduced the glomerular cross-sectional area (*n* ≥ 3 mice/group). (**G**) Under untreated conditions, the p-ERK level in the kidneys of *Cdh16-Cre*; *Nphp3^flox/flox^* mice was significantly higher than that in *Nphp3^flox/flox^* mice. The administration of CI-1040 (10 mg/kg) led to a significant reduction in renal phospho-ERK (p-ERK) levels in *Cdh16-Cre*; *Nphp3^flox/flox^* mice. The proportion of p-ERK-positive cells was significantly reduced following CI-1040 treatment (*n* = 3 mice/group). Statistical significance was defined as * *p* < 0.05; *** *p* < 0.001; and *p* > 0.05 (NS [not significant]) using an unpaired two-tailed Student’s *t*-test for two-group comparisons.

The nephron segment in which renal cysts formed in mice with a *Cdh16-Cre*-mediated *Nphp3* deletion, which is known to drive recombination predominantly in the distal convoluted tubules (DCT) and collecting ducts (CD), was determined by performing segment-specific immunofluorescence using *Lotus tetragonolobus* lectin (LTL-FITC) for the proximal tubules, SLC12A3-Cy3 for the DCT, and AQP2-Cy5 for the CD. As shown in Figure 5A, cystic structures consistently colocalized with SLC12A3 and AQP2 signals but were negative for LTL signals, confirming that cystogenesis arose exclusively from Cdh16-Cre-targeted segments.

#### 2.2.2. Renal Tubular Epithelial Cells in *Cdh16-Cre*; *Nphp3^flox/flox^* Mice Exhibited Proliferation, Polarity Abnormalities, and Injury

In this study, we performed staining for the cell proliferation marker Ki-67 and found that compared with that in the control mice, the number of Ki-67-positive renal tubular cells was significantly increased in *Cdh16-Cre*; *Nphp3^flox/flo^^x^* mice (Figure 5C). Simultaneously, we conducted immunofluorescence staining for zonula occludens-1(ZO-1), a marker that reflects tubular cell polarity and epithelial integrity. The results indicated that renal tubular epithelial cells, particularly those lining the cysts, exhibited polarity abnormalities and disrupted integrity in *Cdh16-Cre*; *Nphp3^flox/flo^^x^* mice (Figure 5B). The gene expression levels of renal tubular injury markers (kidney injury molecule-1[KIM-1] and neutrophil gelatinase-associated lipocalin [NGAL]) were examined in 1 and 2-week-old mice. The results showed that the expression of these tubular injury markers was significantly upregulated in *Cre*; *Nphp3^flox/flox^* mice (Figure 5D).

#### 2.2.3. Renal Cysts in *Cdh16-Cre*; *Nphp3^flox/flox^* Mice Can Be Rescued by Tolvaptan and CI-1040

We assessed whether the cystic phenotype of our tubule-specific *Nphp3* knockout model mice was driven by pathogenic mechanisms shared with other forms of polycystic kidney disease and thus was amenable to targeted therapeutic intervention by evaluating the efficacy of two well-established pathway inhibitors: tolvaptan, a selective vasopressin V2 receptor (V2R) antagonist, and CI-1040, a mitogen-activated extracellular signal-regulated kinase (MEK) inhibitor. Both agents have previously shown efficacy in attenuating cystogenesis in the *Nphp3^pcy/pcy^* model, implicating dysregulated cAMP signaling and hyperactivated mitogen-activated protein kinase/extracellular signal-regulated kinase (MAPK/ERK) pathways in *Nphp3*-related cyst formation [8,9]. In this study, we measured cAMP levels in the kidneys of 1 and 2-week-old mice. Figure 5E shows that an increasing trend in cAMP levels was already observable at 1 week, which became more pronounced at 2 weeks. Additionally, we assessed phospho-ERK (p-ERK) levels in the kidneys of 2-week-old mice and observed a significant upregulation of p-ERK in *Cdh16-Cre*; *Nphp3^flox/flox^* mice (Figure 5G). In the present study, *Cdh16-Cre*; *Nphp3^flox/flox^* mice were administered tolvaptan (5 mg/kg/day) or CI-1040 (10 mg/kg/day) daily from postnatal days 5 to 14. At 2 weeks of age, both treatments significantly reduced the kidney-to-body weight ratio (Figure 5F) and markedly suppressed renal cyst development, as evidenced by the histological analysis of H&E-stained kidney sections (Figure 5F). These findings confirm that the core cystogenic pathways disrupted in classical polycystic kidney disease remain operative in this nephronophthisis model and validate *Cdh16-Cre*; *Nphp3^flox/flox^* mice as a physiologically relevant platform for preclinical testing of mechanism-based therapeutics.

## 3. Discussion

In this study, a renal tubule-specific *Nphp3* knockout mouse model was established. The renal phenotype of this model is consistent with that observed in NPHP3 patients, and is characterized by renal cyst formation, renal fibrosis, rapid progression of renal dysfunction, and a shortened lifespan (5–8 weeks). While systemic development appeared normal in the early stages, body weight gain became restricted during the mid-to-late phases, with weight loss and emaciation observed near the endpoint. Renal cysts in the mice emerged at 1 week of age and progressively enlarged thereafter, compressing the surrounding renal parenchyma. Quantification of the number and cross-sectional area of glomeruli in maximal renal sections revealed a gradual decrease in the glomerular count, accompanied by compensatory hypertrophy in the remaining glomeruli at later stages. Immunofluorescence staining indicated that the renal cysts originated from the distal tubules and collecting ducts. In the model mice, cysts may originate from the proliferation of tubular epithelial cells, accompanied by damage to the integrity of the renal tubular epithelium. This spatial concordance between Cre-expressing domains and cyst formation underscores that the loss of Nphp3 specifically in the distal nephron is sufficient to initiate cystic transformation, aligning the cellular phenotype with the genetic targeting strategy. Cyst formation could be rescued by treatment with tolvaptan and CI-1040. Furthermore, renal fibrosis began at 2 weeks and progressively worsened over time. Renal immune cell infiltration began at 1 week of age, preceding the period of overt renal cyst formation. This suggests that fibrosis in this mouse model is a process independent of cystogenesis and is likely associated with the loss of Nphp3 expression in renal tubules. In contrast to the clinical manifestations in patients, the histopathological examination of other major organs and liver function tests in this kidney-specific mouse model revealed no abnormalities.

NPHP-RCs are a group of hereditary progressive tubulointerstitial kidney diseases that frequently affect ciliated organs such as the kidneys, liver, and brain [1]. Currently, seven *Nphp3*-related mouse models have been established [13], among which the most commonly used is the *Nphp3^pcy/pcy^* mouse [14]. This model was initially employed as a polycystic kidney disease model and results from a spontaneous missense mutation (c.1823T > C) in the *Nphp3* gene. It is characterized by progressively expanding cysts in the renal collecting ducts and biliary system. Cystic lesions can be observed in neonatal mice of this model. In vivo changes in renal T1 relaxation time (T1) and T1 relaxation time (T2) values can be detected by magnetic resonance imaging (MRI) in *Nphp3^pcy/pcy^* mice as early as 4–5 weeks of age [15]. However, significant kidney enlargement does not occur until 8 weeks of age, and azotemia develops after 18 weeks. Renal cysts in this model originate from all segments of the nephron and the collecting ducts [16]. *NPHP3* gene deficiency leads to ciliary dysfunction and a disruption of cell polarity, which are critical contributors to renal cystogenesis [17]. Subsequent studies of the *Nphp3^pcy/pcy^* model have shown that cyst formation is associated with elevated levels of p-ERK and cAMP and that the cystic phenotype can be rescued by treatment with tolvaptan and CI-1040 [11,12]. Furthermore, a high-protein diet exacerbated NPHP similarly; in the mouse model used in this study, cyst formation was also rescued by tolvaptan and CI-1040, highlighting a shared mechanism between the two models.

In contrast to the *Nphp3^pcy/pcy^* model, the model used in this study specifically exhibits targeted knockout of exons 2–3 of the *Nphp3* gene in the kidney tubules; exon 1 was excluded because of its overlap with other genes. A global knockout approach was not adopted because it leads to embryonic lethality [8], precluding the generation of viable mice. *Cdh16-Cre*; *Nphp3^flox/flox^* mice exhibit more rapid progression of renal dysfunction and a shorter lifespan, which is consistent with the accelerated disease progression reported in previous clinical studies of NPHP3 patients [4]. This shorter survival period, combined with a defined therapeutic window, allows for shorter experimental cycles in this model. Additionally, the absence of pathology in other major organs in this model provides an advantage for studies focused exclusively on kidney disease.

Based on previous studies of the *Nphp3^pcy/pcy^* mouse model, the mechanisms underlying cyst formation primarily include increased cAMP levels and the activation of the MAPK/ERK, mTOR and Hippo pathways [2,18,19,20,21]. In *Nphp3^pcy/pcy^* mice, the knockout of periostin effectively reduces renal cyst formation and progression [22]. The administration of rapamycin decreases the kidney size [23]. Dietary phosphate restriction in *Nphp3^pcy/pcy^* mice slows cystogenesis [24]. The inhibition of cAMP production via octreotide treatment rescues the percentage of abnormal spheroids [17]. Additionally, the administration of tolvaptan, a calcimimetic, or CI-1040 to *Nphp3^pcy/pcy^* mice suppresses cyst formation and development [20,25,26,27]. In the present study, we also selected two classical pathways for intervention. The administration of tolvaptan and CI-1040 reduced the number of cysts, confirming the similarity of the pathogenic mechanisms between the model used in our study and the *Nphp3^pcy/pcy^* mouse model.

However, *Cdh16-Cre*; *Nphp3^flox/flox^* mice lack extrarenal phenotypic manifestations, particularly the hepatic manifestations commonly observed in NPHP3 patients. Furthermore, the rapid disease progression in this mouse model results in a narrow therapeutic window for intervention. The limited intervention strategies available for early postnatal mice also pose significant challenges for pharmacological studies. Most importantly, this model cannot fully recapitulate the complexity of renal microenvironment interactions or the systemic involvement that is a characteristic of the human disease. Furthermore, a limitation shared with other existing animal models is the inability to recapitulate the diverse clinical phenotypes observed in human diseases caused by *NPHP3* mutations, such as other clinical syndromes (e.g., Meckel syndrome 7, Renal-hepatic-pancreatic dysplasia 1). Additionally, as the mouse model utilized in this study employs the Flox-Cre system, Cre activation cannot be induced during adulthood. Consequently, the model is unable to detect phenotypes that manifest only in adulthood. Due to the lack of direct measurements of glomerular functional parameters in this study, we can only infer that the renal function impairment in mice primarily originates from tubular damage, without being able to exclude the potential contribution of glomerular involvement.

In summary, we established a renal tubule-specific *Nphp3* knockout mouse model. Compared with the conventional *Nphp3^pcy/pcy^* model, this model better recapitulates the phenotypic features of NPHP3 patients, particularly in terms of rapid disease progression. Furthermore, the use of this model offers shorter experimental timelines and greater suitability for kidney-specific research by eliminating confounding phenotypes from other organs.

## 4. Materials and Methods

### 4.1. Generation and Characterization of Cdh16-Cre; Nphp3^flox/flox^ Mice

#### 4.1.1. Generation of *Nphp3^flox/+^* Mice

We generated floxed *Nphp3* alleles using CRISPR/Cas9-mediated homologous recombination in mouse zygotes. We transcribed Cas9 mRNA and gRNA in vitro and constructed a homologous donor vector using In-Fusion cloning. This vector featured a 1.5 kb floxed sequence flanked by 2.9 kb 5′ and 2.7 kb 3′ homology arms. We then microinjected the Cas9 mRNA, gRNA, and donor vector into C57BL/6J mouse zygotes to produce F0 founders. After genotyping by polymerase chain reaction (PCR) and sequencing, positive Filial 0 (F0) mice were bred with C57BL/6J mice, resulting in the *Nphp3^flox/+^* (F1) generation.

#### 4.1.2. Generation of *Cdh16-Cre*; *Nphp3^flox/+^* Mice

We divided the heterozygous floxed (*Nphp3^flox/+^*) mice into two groups. The first cohort was bred with Cre-driver mice to yield progeny that were either positive for both the floxed allele and Cre (*Cdh16-Cre*; *Nphp3^flox/+^*) or positive for the floxed allele alone (*Nphp3^flox/flox^*). The use of the Cdh16-Cre driver line ensured that Cre recombinase activity was predominantly restricted to renal tubular cells, including the loop of Henle, the distal tubule, the collecting duct, the proximal tubule, and Bowman’s capsule. The second cohort was intercrossed to generate homozygotes (*Nphp3^flox/flox^*). The double-positive heterozygous *Cdh16-Cre*; *Nphp3^flox/+^* mice were then crossed with homozygous floxed *Nphp3^flox/flox^* mice to obtain *Cdh16-Cre*; *Nphp3^flox/flox^* mice for further study.

### 4.2. Animal Studies

*Cdh16-Cre*; *Nphp3^flox/+^* and *Nphp3^flox/+^* mice (with specific ages detailed in the respective results) were supplied by Shanghai Model Organisms (Shanghai, China). All animal procedures were conducted in accordance with the institutional guidelines for the care and use of laboratory animals. Tolvaptan (MCE, HY-50901, Princeton, NJ, USA) was initially dissolved in DMSO to prepare a stock solution. This stock solution was subsequently diluted in a vehicle consisting of 40% PEG 300, 5% Tween-80, and 45% saline for administration. Tolvaptan was administered via intraperitoneal injection at a dosage of 5 mg/kg once daily from postnatal day 5 (P5) to postnatal day 14 (P14).

As urine collection using metabolic cages (TSE Systems, Berlin, Germany) was not feasible for 1–2-week-old mice, spontaneously voided random urine samples were collected for analyses. Blood samples were obtained from the retro-orbital venous plexus. The blood was placed in pro-coagulation tubes, allowed to clot, and then centrifuged to isolate serum.

Following euthanasia via CO_2_ asphyxiation, the organs were intracardially perfused with phosphate-buffered saline (PBS) (Thermo Fisher, 10010023, Waltham, MA, USA) to flush the blood from the organs. The required organs were then harvested for subsequent studies.

### 4.3. Genotyping

The collected mouse tails were placed in corresponding labeled eppendorf (EP) tubes. A total of 200 µL of SNET lysis buffer and 20 µL of proteinase K solution (final concentration: 200 µg/mL) were added to each tube. The samples were incubated overnight at 60 °C until complete tissue dissolution occurred.

Following digestion, 400 µL of absolute ethyl alcohol was added to each tube and mixed thoroughly. White flocculent precipitates became visible after mixing. The tubes were subsequently centrifuged at 12,000 rpm for 10 min at room temperature. The supernatant was discarded, and the pellet was washed with 500 µL of 75% ethanol. After thorough washing, the samples were centrifuged again at 12,000 rpm for 5 min. The supernatant was carefully removed, and the pellets were air-dried for 10 min with the tube caps open. Finally, each pellet was resuspended in 50–100 µL of sterile distilled water to obtain genomic deoxyribonucleic acid (DNA) samples.

The DNA samples were subjected to PCR using the primer sets listed below. For the identification of the floxed allele, P1 (5′→3′: TCTCAGCTGTCAGGACATCATC) and P2 (5′→3′: AAATGGAGAATAGGTACTTTGGGT) were used. For Cdh16-Cre transgene identification, P3 (5′→3′: GCAGATCTGGCTCTCCAAAG), P4 (5′→3′: AGGCAAATTTTGGTGTACGG), P5 (5′→3′: CAAATGTTGCTTGTCTGGTG), and P6 (5′→3′: GTCAGTCGAGTGCACAGTTT) were used. The detailed PCR components and cycling conditions are provided in Supplementary Table S1.

Genotypes were determined by analyzing the PCR fragment patterns. The presence of the flox allele was discerned by the amplification of either a 285 bp product (wild-type allele) or a 354 bp product (floxed allele), resulting in a single 285 bp band for wild-type (WT) mice, both a 354 bp and a 285 bp band for heterozygotes, and a single 354 bp band for homozygotes. Moreover, the Cdh16-Cre transgene was identified by the presence of an approximately 420 bp amplification product, with its absence indicated by a 200 bp control band.

### 4.4. Assessment of CRE Recombinase Activity

Tissue samples from the relevant organs were dissected and placed in 1.5 mL EP tubes. Genomic DNA was extracted from these tissues using the method described above for genotyping. The DNA was subsequently subjected to PCR amplification with primers specific for the recombined locus (P7: 5′-AAGCTGTTCTCACCCTCAGC-3′; P8: 5′-TGTTCCATGACAGGTTGGGG-3′). The detailed PCR mixture and thermal cycling conditions are provided in Supplementary Table S1. The PCR products were separated by electrophoresis on a 1% agarose gel at 150 V for 30 min. A product of 907 bp indicates the presence of Cre-mediated recombination and thus successful Cre activity, whereas the amplification of a 2463 bp fragment indicates the absence of recombination.

### 4.5. Measurement of Liver and Kidney Function

We collected whole blood from the retro-orbital venous plexus and allowed it to clot in an Eppendorf tube, followed by centrifugation. Serum creatine levels were measured using the sarcosine oxidase method, serum urea nitrogen levels were measured using the urease–glutamate dehydrogenase method, and alanine aminotransferase (ALT) levels and aspartate aminotransferase (AST) levels were measured using the aspartate substrate method. Alkaline phosphatase (ALP) activity was measured with the p-nitrophenyl phosphate method, total bilirubin (TBIL) activity was measured with the vanadic acid oxidation method, direct bilirubin (DBIL) activity was measured with the diazo reagent method, serum urea (UREA) activity was measured with the urease–glutamate dehydrogenase method, and serum creatinine (SCR) activity was measured with the sarcosine oxidase method.

### 4.6. Western Blot

Protein samples were obtained from the kidneys of postnatal day 0, 7, and 14 mice. Renal tissue was homogenized in 1 mL of precooled radio-immunoprecipitation assay (RIPA) lysis buffer (Beyotime, P0013B, Shanghai, China) containing protease and phosphatase inhibitors (Beyotime, P1005; Beyotime, P1045). Three 3 mm zirconium oxide grinding beads (Servicebio, G0203-150G, Wuhan, China) were added, and the mixture was ground in a precooled homogenizer (2 min, 60 Hz). The lysate was incubated on ice for 30 min, followed by centrifugation at 4 °C and 12,000 rpm for 20 min. The supernatant was transferred to a clean EP tube, and a 1/4 volume of 5× loading buffer (Beyotime, P0015, Shanghai, China) was added. The mixture was thoroughly blended and heated at 95 °C for 5–10 min to denature the proteins. Electrophoresis was performed on a 10% polyacrylamide gel (ACE Biotechnology, ET15008L Gell, Changzhou, China) at 100 V for 2 h. Proteins were transferred to a 0.45 µm polyvinylidene fluoride (PVDF) (Beyotime, FFP24) membrane at 360 mA for 90 min. The membrane was blocked with 5% skim milk for 30 min and then washed three times with phosphate-buffered saline containing 0.1% Tween 20 (PBST) (10 min each). The primary antibodies used were against GAPDH (Proteintech, Cat No.: 60004-1-Ig, dilution 1:1000, Wuhan, China), fibronectin (Proteintech, Cat No.: 15613-1-AP, dilution 1:1000), and α-SMA (Proteintech, Cat No.: 14395-1-AP, dilution 1:1000). The secondary antibody was horseradish peroxidase-labeled goat anti-rabbit IgG (Beyotime, Cat No.: A0208, dilution 1:1000). Detection was performed using a hypersensitive ECL chemiluminescence kit (Beyotime, P0018M). Band intensities were analyzed with ImageJ software (ImageJ 1.53t), and intergroup comparisons were conducted.

### 4.7. Quantitative Real-Time PCR

RNA was extracted from mouse kidneys using TRIzol (Life Technologies, Carlsbad, CA, USA). The RNA quality was evaluated by measuring the ratio of the optical density at 260 nm/280 nm. RNA (~1 µg) was reverse transcribed to cDNA using the Prime Script RRT kit (TaKaRa, Beijing, China) according to the manufacturer’s instructions. Amplification was performed with AceQ qPCR SYBR Green Master Mix (Vazyme, Nanjing, China) and a real-time qPCR system (Agilent Mx3000P, Santa Clara, CA, USA). *Gapdh* was used as an internal control. All primer sequences are provided in Supplementary Table S2. All primers were synthesized by Sangon (Shanghai, China). The results were analyzed using the 2^−ΔΔCt^ method to determine the relative gene expression levels. Each group contained at least three samples, each of which was assayed three times independently.

### 4.8. Histological Analyses

H&E staining was performed on 5 µm-thick paraffin sections according to the standard protocol described in the literature [28]. Following deparaffinization and rehydration as described for H&E staining, the tissue sections were subjected to Masson’s trichrome staining. Briefly, the nuclei were stained with Weigert’s iron hematoxylin for 5–10 min, followed by differentiation in 1% acid alcohol and bluing under running water. The sections were then counterstained with Biebrich scarlet–acid fuchsin for 5–10 min to visualize the cytoplasm and muscle fibers. Subsequent treatment with a 1% phosphomolybdic acid solution for 3–5 min facilitated the differential binding of aniline blue, which was applied directly for 3–5 min to selectively stain collagen fibers. Finally, the sections were rinsed with 1% acetic acid, dehydrated through a graded ethanol series, cleared using xylene, and mounted with a neutral resinous medium.

### 4.9. Immunofluorescence Staining and Immunohistochemical Staining

Kidney sections (5 µm) from postnatal day 0, 7, and 14 mice were stained. Following deparaffinization, rehydration, and antigen retrieval, the sections were blocked at room temperature for 30 min with 10% donkey serum.

The sections were subsequently incubated with primary antibodies overnight at 4 °C. Afterward, the sections were washed three times with PBS (5 min each) and counterstained with 4’,6-diamidino-2-phenylindole (DAPI) for 10 min. Following another three washes with PBS (5 min each), the sections were mounted with anti-fade mounting medium. Images were subsequently acquired.

The staining procedure for immunohistochemistry was similar to that for immunofluorescence. Chromogenic detection was performed using HRP-conjugated secondary antibodies, followed by the addition of substrate (DAB) for color development. The reaction was monitored under a microscope and terminated with PBS once the optimal staining intensity was achieved. Subsequently, hematoxylin was used for nuclear counterstaining. After a brief staining period, sections were rinsed under running water, followed by differentiation in hydrochloric acid-alcohol and bluing to enhance cellular structural clarity. The sections were then dehydrated through a graded alcohol series, cleared in xylene, and mounted with anti-fade mounting medium. A coverslip was applied, taking care to avoid air bubbles. The stained sections were subsequently observed and imaged using a microscope (ZEISS, LSM 780, Oberkochen, Germany).

The primary antibodies used were as follows: NPHP3 (Proteintech, Cat No.: 22026-1-AP, dilution 1:200), p-ERK (Proteintech, Cat No.: 80031-1-RR, dilution 1:200), SLC12A3-CY3 (Affinity, Cat No.: AF5101, dilution 1:100, Changzhou, China), LTL-FITC (Vector Labs, Cat No.: B-1325, dilution 1:50, Newark, CA, USA), and AQP2-CY5 (Affinity, Cat No.: DF7560, dilution 1:100), CD11B-CY3 (abcam, ab133357, dilution 1:1000, Cambridge, UK), ZO-1 (Proteintech, Cat No.: 21773-1-AP, dilution 1:200), and Ki-67 (HuaBio, Cat No.: 28074-1-AP, dilution 1:200, Hangzhou, China). After the incubation, the sections were washed three times with PBS (5 min each). The sections were subsequently incubated with secondary antibodies for 1 h at room temperature in the dark. The secondary antibodies used were HRP-conjugated goat anti-rabbit (SeraCare, Cat: 5220–0336, dilution 1:400, Milford, MA, USA), HRP-conjugated goat anti-mouse (SeraCare, Cat: 5220–0341, dilution 1:400), 488-goat anti-rabbit recombinant secondary antibody (H + L) (Proteintech, Cat No.: RGAR002, dilution 1:400).

The quantitative analysis of the fluorescence intensity and the number of positively stained cells was performed using ImageJ software, and intergroup comparisons were conducted.

### 4.10. cAMP Level Detection in Mouse Kidneys

The assay was performed using a cAMP ELISA Kit (Sangon Biotech, Cat No.: D770001-0096, Shanghai, China), which is based on the competitive enzyme immunoassay principle. Prior to the experiment, the kit components were equilibrated to room temperature. Freshly collected kidney tissues were snap-frozen in liquid nitrogen and stored until use. For analysis, the frozen samples were weighed. And the subsequent steps were carried out in accordance with the manufacturer’s instructions. A standard curve was generated for quantification of cAMP levels.

### 4.11. Image Analysis

Glomerular quantification and area morphometry were performed with Case Viewer 2.4 using digital scans of the stained tissue sections.

### 4.12. Silver Staining of Urine Proteins

Random urine samples from mice were collected and clarified by centrifugation at 1000 rpm for 5 min. The supernatant was then mixed with a one-quarter volume of 5× loading buffer and denatured at 95 °C for 5 min. Electrophoresis was performed on a 10% polyacrylamide gel (ACE Biotechnology, ET15008LGell, Changzhou, China) at 100 V for 2 h. The gel was subsequently subjected to silver staining using a Fast Silver Stain Kit (Beyotime, P0017S).

### 4.13. Statistics

The survival of the mice was analyzed using the Kaplan—Meier method, and differences in survival curves among different genotypes or treatment groups were compared using the log-rank test. Other data were processed using GRAPHPAD PRISM 8.0 statistical software. The data are presented as the means ± SEMs. Differences between the two groups were determined using unpaired *t* tests or Fisher’s exact test. The significance level was set at *p* < 0.05.

## Data Availability

The raw data supporting the conclusions of this article will be made available by the authors on request.

## Supplementary Materials

The following supporting information can be downloaded at https://www.mdpi.com/article/10.3390/ijms27062687/s1.

## References

[1] Otto E.A., Ramaswami G., Janssen S., Chaki M., Allen S.J., Zhou W., Airik R., Hurd T.W., Ghosh A.K., Wolf M.T. (2011). Mutation analysis of 18 nephronophthisis associated ciliopathy disease genes using a DNA pooling and next generation sequencing strategy. J. Med. Genet..

[2] Hildebrandt F., Attanasio M., Otto E. (2009). Nephronophthisis: Disease mechanisms of a ciliopathy. J. Am. Soc. Nephrol. JASN.

[3] Wolf M.T.F., Bonsib S.M., Larsen C.P., Hildebrandt F. (2024). Nephronophthisis: A pathological and genetic perspective. Pediatr. Nephrol..

[4] Tang X., Liu C., Liu X., Chen J., Fan X., Liu J., Ma D., Cao G., Chen Z., Xu D. (2022). Phenotype and genotype spectra of a Chinese cohort with nephronophthisis-related ciliopathy. J. Med. Genet..

[5] Braun D.A., Hildebrandt F. (2017). Ciliopathies. Cold Spring Harb. Perspect. Biol..

[6] Sakakibara N., Nozu K., Yamamura T., Horinouchi T., Nagano C., Ye M.J., Ishiko S., Aoto Y., Rossanti R., Hamada R. (2022). Comprehensive genetic analysis using next-generation sequencing for the diagnosis of nephronophthisis-related ciliopathies in the Japanese population. J. Hum. Genet..

[7] Luo F., Tao Y.H. (2018). Nephronophthisis: A review of genotype-phenotype correlation. Nephrology.

[8] Bergmann C., Fliegauf M., Brüchle N.O., Frank V., Olbrich H., Kirschner J., Schermer B., Schmedding I., Kispert A., Kränzlin B. (2008). Loss of nephrocystin-3 function can cause embryonic lethality, Meckel-Gruber-like syndrome, situs inversus, and renal-hepatic-pancreatic dysplasia. Am. J. Hum. Genet..

[9] Olbrich H., Fliegauf M., Hoefele J., Kispert A., Otto E., Volz A., Wolf M.T., Sasmaz G., Trauer U., Reinhardt R. (2003). Mutations in a novel gene, NPHP3, cause adolescent nephronophthisis, tapeto-retinal degeneration and hepatic fibrosis. Nat. Genet..

[10] Zhou W., Dai J., Attanasio M., Hildebrandt F. (2010). Nephrocystin-3 is required for ciliary function in zebrafish embryos. Am. J. Physiol. Ren. Physiol..

[11] Frank V., Habbig S., Bartram M.P., Eisenberger T., Veenstra-Knol H.E., Decker C., Boorsma R.A.C., Göbel H., Nürnberg G., Griessmann A. (2013). Mutations in NEK8 link multiple organ dysplasia with altered Hippo signalling and increased c-MYC expression. Hum. Mol. Genet..

[12] Boehlke C., Kotsis F., Patel V., Braeg S., Voelker H., Bredt S., Beyer T., Janusch H., Hamann C., Gödel M. (2010). Primary cilia regulate mTORC1 activity and cell size through Lkb1. Nat. Cell Biol..

[13] Mouse Genome Informatics. Nphp3. https://www.informatics.jax.org/quicksearch/summary?queryType=exactPhrase&query=nphp3&submit=Quick+Search.

[14] Takahashi H., Ueyama Y., Hibino T., Kuwahara Y., Suzuki S., Hioki K., Tamaoki N. (1986). A new mouse model of genetically transmitted polycystic kidney disease. J. Urol..

[15] Towner R.A., Yamaguchi T., Philbrick D.J., Holub B.J., Janzen E.G., Takahashi H. (1991). In vivo proton magnetic resonance imaging and localized spectroscopic analysis of polycystic kidney disease in DBA2FG-pyc mice. Magn. Reson. Imaging.

[16] Takahashi H., Calvet J.P., Dittemore-Hoover D., Yoshida K., Grantham J.J., Gattone V.H. (1991). A hereditary model of slowly progressive polycystic kidney disease in the mouse. J. Am. Soc. Nephrol..

[17] Ghosh A.K., Hurd T., Hildebrandt F. (2012). 3D spheroid defects in NPHP knockdown cells are rescued by the somatostatin receptor agonist octreotide. Am. J. Physiol. Ren. Physiol..

[18] Stokman M.F., Saunier S., Benmerah A. (2021). Renal Ciliopathies: Sorting Out Therapeutic Approaches for Nephronophthisis. Front. Cell Dev. Biol..

[19] Gattone V.H., Wang X., Harris P.C., Torres V.E. (2003). Inhibition of renal cystic disease development and progression by a vasopressin V2 receptor antagonist. Nat. Med..

[20] Omori S., Hida M., Fujita H., Takahashi H., Tanimura S., Kohno M., Awazu M. (2006). Extracellular signal–regulated kinase inhibition slows disease progression in mice with polycystic kidney disease. J. Am. Soc. Nephrol..

[21] Parnell S.C., Raman A., Zhang Y., Daniel E.A., Dai Y., Khanna A., Reif G.A., Vivian J.L., Fields T.A., Wallace D.P. (2022). Expression of active B-Raf proto-oncogene in kidney collecting ducts induces cyst formation in normal mice and accelerates cyst growth in mice with polycystic kidney disease. Kidney Int..

[22] Wallace D.P., White C., Savinkova L., Nivens E., Reif G.A., Pinto C.S., Raman A., Parnell S.C., Conway S.J., Fields T.A. (2014). Periostin promotes renal cyst growth and interstitial fibrosis in polycystic kidney disease. Kidney Int..

[23] Gattone V.H., Sinders R.M., Hornberger T.A., Robling A.G. (2009). Late progression of renal pathology and cyst enlargement is reduced by rapamycin in a mouse model of nephronophthisis. Kidney Int..

[24] Omede F., Zhang S., Johnson C., Daniel E., Zhang Y., Fields T.A., Boulanger J., Liu S., Ahmed I., Umar S. (2020). Dietary phosphate restriction attenuates polycystic kidney disease in mice. Am. J. Physiol. Ren. Physiol..

[25] Lee J.W., Cho J.Y., Thuy P.X., Moon E.Y. (2022). HeLa Cervical Cancer Cells Are Maintained by Nephronophthisis 3-Associated Primary Cilium Formation via ROS-Induced ERK and HIF-1α Activation under Serum-Deprived Normoxic Condition. Int. J. Mol. Sci..

[26] Torres V.E., Chapman A.B., Devuyst O., Gansevoort R.T., Grantham J.J., Higashihara E., Perrone R.D., Krasa H.B., Ouyang J., Czerwiec F.S. (2012). Tolvaptan in patients with autosomal dominant polycystic kidney disease. New Engl. J. Med..

[27] Chen N.X., Moe S.M., Eggleston-Gulyas T., Chen X., Hoffmeyer W.D., Bacallao R.L., Herbert B.S., Gattone V.H. (2011). Calcimimetics inhibit renal pathology in rodent nephronophthisis. Kidney Int..

[28] Du X., Yu M., Ju H., Xue S., Li Y., Wu X., Xu H., Shen Q. (2023). Inhibition of MAPK/ERK pathway activation rescues congenital anomalies of the kidney and urinary tract (CAKUT) in Robo2^PB/+^ Gen1^PB/+^ mice. Biochem. Biophys. Res. Commun..

