# Supplementary material for: Renal Tubule-Specific Deletion of Nephrocystin 3 (Nphp3) Causes Infantile Nephronophthisis-like Phenotypes in Mice
Source: Int J Mol Sci. 2026 Mar 15;27(6):2687. doi: 10.3390/ijms27062687 (PMC13026956; doi:10.3390/ijms27062687)
Supplement: Supplementary file 1 [file ijms-27-02687-s001.zip › supplement 1.pdf]

Table S1: Mouse genotype identification and CRE enzyme activity primers and PCR procedures.

Nphp3-Flox

<table>
<tr><td>Primer</td><td colspan="3">Sequence 5' --> 3'</td><td>Primer type</td></tr>
<tr><td>P1/6963</td><td colspan="3">TCTCAGCTGTCAGGACATCATC</td><td>Forward</td></tr>
<tr><td>P2/6940</td><td colspan="3">AAATGGAGAATAGGTACTTTGGGT</td><td>Reverse</td></tr>
<tr><td rowspan="8">PCR Reaction System</td><td colspan="3">Reaction Component</td><td>Volume (μl)</td></tr>
<tr><td colspan="3">ddH2O</td><td>7.0</td></tr>
<tr><td colspan="3">2x transTaq-T PCR SuperMix *</td><td>10.0</td></tr>
<tr><td colspan="3">P1 (10pmol/μl)</td><td>0.5</td></tr>
<tr><td colspan="3">P2 (10pmol/μl)</td><td>0.5</td></tr>
<tr><td colspan="3">Genomic DNA（50-100ng/μl）</td><td>2</td></tr>
<tr><td colspan="3">Total</td><td>20</td></tr>
<tr><td colspan="4">*2×transTaq-T PCR SuperMix from TransGen Biotech (Code number: AS122)</td></tr>
<tr><td rowspan="7">Cycling Reaction</td><td>Step</td><td>Temp (℃)</td><td>Time</td><td>Note</td></tr>
<tr><td>1</td><td>94</td><td>3 min</td><td rowspan="3"></td></tr>
<tr><td>2</td><td>94</td><td>30 sec</td></tr>
<tr><td>3</td><td>60</td><td>30 sec</td></tr>
<tr><td>4</td><td>72</td><td>30 sec</td><td>repeat steps 2-4 for 34cycles</td></tr>
<tr><td>5</td><td>72</td><td>5 min</td><td rowspan="2"></td></tr>
<tr><td>6</td><td>12</td><td>Hold</td></tr>
<tr><td>Result</td><td colspan="4">PCR Products:
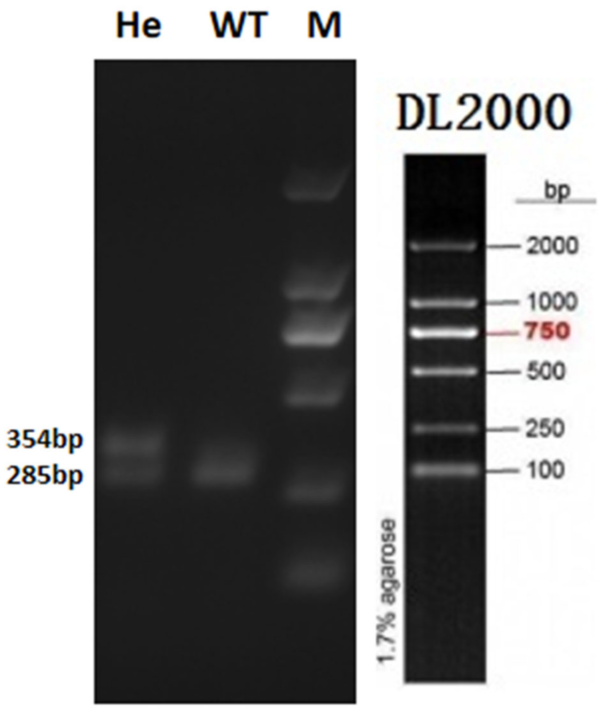

Separated by gel electrophoresis on a 2 % agarose gel.</td></tr>
<tr><td>Genotype</td><td colspan="4">WT: one band with 285 bp;</td></tr>
</table>

	Heterozygous: two bands with 354 and 285 bp;<br>Homozygous: one band with 354 bp;

Cdh16-Cre

**Primer**	**Sequence 5' --> 3'**			**Primer type**
**P3/1209**	GCAGATCTGGCTCTCCAAAG			Forward
**P4/1210**	AGGCAAATTTTGGTGTACGG			Reverse
**P5/control-F**	CAAATGTTGCTTGTCTGGTG			Forward
**P6/control-R**	GTCAGTCGAGTGCACAGTTT			Reverse
**PCR Reaction System**	**Reaction Component**			**Volume (µl)**
	ddH2O			6.0
	2x Taq Plus Master Mix *			10.0
	P3 (10pmol/µl)			0.5
	P4 (10pmol/µl)			0.5
	P5 (10pmol/µl)			0.5
	P6 (10pmol/µl)			0.5
	Genomic DNA（50-100ng/µl）			2
	Total			20
	*2×Taq Plus Master Mix from Vazyme (Code number:P212)			
**Cycling Reaction**	**Step**	**Temp (℃)**	**Time**	**Note**
	1	94	3 min	
	2	94	30 sec	
	3	60	30 sec	
	4	72	30 sec	repeat steps 2-4 for 34cycles
	5	72	5 min	
	6	12	Hold	
**Genotype**	Tg: ~420 bp;<br>Control: 200bp;			

Cre activity

**Primer**	**Sequence 5' --> 3'**	**Primer type**
**P7**	AAGCTGTTCTCACCCTCAGC	Forward
**P8**	TGTTCCATGACAGGTTGGGG	Reverse
**PCR Reaction System**	**Reaction Component**	**Volume (µl)**
	ddH2O	8.05
	2xPCR Buffer	10
	Primer V (20pmol/µl)	0.3
	Primer VI (20pmol/µl)	0.3
	KOD-Multi&Epi-*	0.35
	Genomic DNA	1

<table>
<tr><td rowspan="2"></td><td colspan="3">Total</td><td>20</td></tr>
<tr><td colspan="4">* KOD-Multi&Epi-（TOYOBO，Code No：KME-101）</td></tr>
<tr><td rowspan="7">Cycling Reaction</td><td>Step</td><td>Temp (℃)</td><td>Time</td><td>Note</td></tr>
<tr><td>1</td><td>94</td><td>3 min</td><td rowspan="3"></td></tr>
<tr><td>2</td><td>98</td><td>20 sec</td></tr>
<tr><td>3</td><td>63</td><td>20 sec</td></tr>
<tr><td>4</td><td>68</td><td>3 min</td><td>repeat steps 2-4 for 35cycles</td></tr>
<tr><td>5</td><td>68</td><td>5 min</td><td rowspan="2"></td></tr>
<tr><td>6</td><td>12</td><td>Hold</td></tr>
<tr><td>Result</td><td colspan="4">PCR Products:

With Cre activity: 907 bp ; no Cre activity: 2463 bp; wild type：2324 bp。

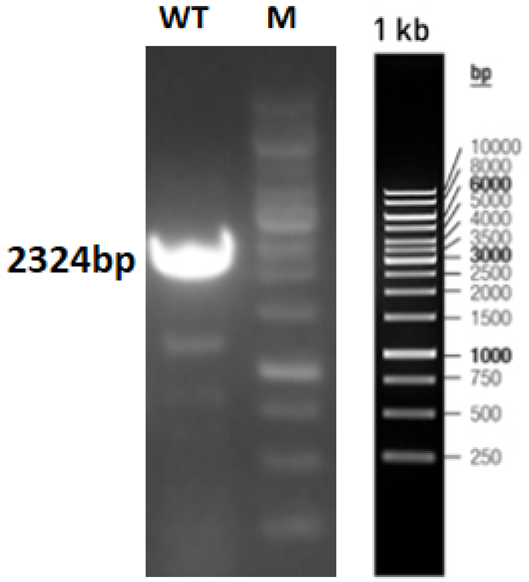

Separated by gel electrophoresis on a 1.0% agarose gel.</td></tr>
</table>
