# Supplementary material for: Renal Tubule-Specific Deletion of Nephrocystin 3 (Nphp3) Causes Infantile Nephronophthisis-like Phenotypes in Mice
Source: Int J Mol Sci. 2026 Mar 15;27(6):2687. doi: 10.3390/ijms27062687 (PMC13026956; doi:10.3390/ijms27062687)
Supplement: Supplementary file 1 [file ijms-27-02687-s001.zip › supplement 2.pdf]

Table S2: Mouse QPCR primers.

gapdh F	AATGGATTTGGACGCATTGGT
gapdh R	TTTGCACTGGTACGTGTTGAT
Fibronectin 1 F	GGAACCTACAGGGGAAGGAAG
Fibronectin 1 R	CCACTGGACCGTCACTGTC
Havcr1 F	ACATATCGTGGAATCACAACGAC
Havcr1 R	ACTGCTCTTCTGATAGGTGACA
Lcn2 F	TGGCCCTGAGTGTCATGTG
Lcn2 R	CTCTTGTAGCTCATAGATGGTGC
